# Determination of thermal transitions of gluten-free chestnut flour doughs enriched with brown seaweed powders and antioxidant properties of baked cookies

**DOI:** 10.1016/j.heliyon.2019.e01805

**Published:** 2019-06-04

**Authors:** Santiago Arufe, Jorge Sineiro, Ramón Moreira

**Affiliations:** Department of Chemical Engineering, Universidade de Santiago de Compostela, rúa Lope Gómez de Marzoa, Santiago de Compostela, E-15782, Spain

**Keywords:** Food science, Chemical engineering, Thermal process calculations in food engineering, Viscoelasticity, Gluten-free food, Starch, Antioxidant additive, Baking

## Abstract

A protocol for determining the characteristic temperatures of thermomechanical transitions on gluten-free flour doughs is proposed. This protocol is based on the mathematical analysis of experimental curve of storage modulus (G′) *vs* temperature obtained by means of Dynamic Mechanical Thermal Analysis (DMTA) technique. Doughs at constant consistency of chestnut flour with different levels (3, 6 and 9% flour basis, f.b.) of brown seaweed (*Bifurcaria bifurcata*, *Fucus vesiculosus* and *Ascophyllum nodosum*) powders addition, 2% f.b. of guar gum and 1.8% f.b. of salt with different water absorption were used to test the proposed protocol. The ranges of temperatures corresponding to starch gelatinization (59–97 °C), amylopectin crystallites melting (82–101 °C), reversible dissociation of lipid-amylose complexes (107–128 °C) and amylose melting (133–171 °C) showed a strong dependence with water absorption of samples. Doughs with the same water absorption submitted to starch gelatinization during mixing were also analysed to corroborate the protocol suitability. Total polyphenols content and radical scavenging activity of extracts from chestnut flour-seaweed powder blends and seaweed-enriched chestnut cookies baked at 180 °C were determined. Extraction assisted with ultrasounds was carried out employing acetone-water (70:30 v/v) solution as solvent during 4 min with a liquid/solid ratio of 30 w/w. Seaweed powder addition had a positive effect on antioxidant properties of doughs before baking. However, the seaweed powder addition effect on baked products (cookies) is not clear due to antioxidant activity is overlapped by Maillard's products generated during baking.

## 1 Introduction

Dough can be defined as a blend of flour and water, obtained by involving mechanical energy to obtain a final mixed product. In bakery products, kneading, together with baking, is one of the most important steps that influence the final product quality [1]. Once a dough is obtained after kneading, the study on the transformations during thermal process promoted during baking is essential to estimate and control the final product properties.

Differential scanning calorimetry (DSC) is the most common method to study the thermal behaviour of isolated starches for determining temperature of transitions and the corresponding enthalpies [2, 3]. Particularly, the starch gelatinization involving the swelling of the amorphous region and subsequent melting of crystallites, usually named as G transition, is well studied by its importance in starch processing for food and non-food purposes. Thermal transitions often depend on water content of the system. For example, G transition is observed as one broad endothermic peak at high water content. However, at intermediate water content the melting of crystallites is partially postponed to higher temperatures in form of another peak known as M1 transition [4, 5]. Thermal transitions different to starch gelatinization and associated to other biopolymer interactions, have been also determined by DSC. At higher temperatures than those associated to gelatinization, a reversible dissociation of lipid-amylose complexes takes place in the range from 100 to 120 °C [6, 7] which is named as M2 transition. At higher temperatures (>140 °C) also amylose melting transition can be observed [4] and is labelled as M3 transition [6]. These transitions also depend on water content of the sample.

Dynamic Mechanical Thermal Analysis (DMTA) is an experimental method in which a sinusoidal force is applied to the sample at fixed angular frequency measuring the stress and strain inside the linear viscoelaticity region (LVER) at constant heating/cooling rate. These characteristics make DMTA a very interesting technique to evaluate thermal and mechanical transitions on starchy systems in major scale than the one employed using DSC. In the last decade DMTA technique was employed to evaluate the starch gelatinization due to the strong structural changes associated to the plasticizing processes promoted by water [8]. In the case of starch gelatinization, the characteristic range of temperatures can be clearly determined by means of respective storage modulus, G′, complex viscosity, G* and damping factor, tanδ, peaks [9, 10]. However, other thermal transitions that take place in starchy systems are often difficult to be determined regarding rheological parameters (G′, G* and tanδ). The absence of a clear protocol for the determination of these transitions from experimental data obtained through DMTA technique often makes the scientific experimentation more complex due to other complementary techniques such as DSC have to be employed. Therefore, the establishment of a protocol for the determination of the characteristic temperatures of these transitions employing DMTA could be very useful. These thermal transitions show a strong water content dependence, consequently the use of different starchy systems with different water content is appropriate to establish and test the new protocol. Also, the study by DMTA of doughs previously submitted to a starch gelatinization process is proposed to corroborate the protocol for the thermal transitions determination.

Given the increasing demand of gluten-free products adequate for people suffering some of the three pathologies associated with the gluten intake (*i.e.* gluten allergy, coeliac disease, and gluten sensitivity), the development of new formulations is necessary. In this sense, the use of alternative gluten-free sources like chestnut flour becomes a very interesting starting point [11]. However, the absence of gluten hinders the gas retention by the dough during fermentation process in order to increase its volume for bread manufacture. Nevertheless, chestnut flour can be adequate to develop gluten-free cookies due to the lower requirements of gas retention of these baked products. Moreover, the development of enriched and healthy gluten-free products has a growing demand by the market. In this sense, brown seaweeds such as *Ascophyllum nodosum*, *Bifurcaria bifurcata* and *Fucus vesiculosus* are underexploited marine raw materials to use in food products because of their high nutritional value as source of dietary fibre and healthy benefits [12, 13]. A challenge for the coming years would be to introduce these and other novel additives with attractive antioxidant properties in gluten-free products, optimising processing conditions and doses of the compounds [14]. The optimisation of processing conditions of new formulations is critical due to the potential interactions promoted by temperature that could take place between starchy matrix and compounds added with the new ingredients. These interactions could modify the properties of the final product such as *i.e.*: texture, colour, flavour or antioxidant activity. The effect of the addition of brown seaweeds as a new raw material in food products on the antioxidant activity and polyphenols content of the gluten-free material before and after baking will be assessed.

The authors propose the use of chestnut flour as starchy source and brown seaweed powders added to this flour at different content to obtain doughs with different water content levels and compositions. The aims of this work are: *i)* to develop a new method to determine thermal transitions by DMTA and *ii)* to study the effect of seaweed powder addition on antioxidant properties of blends before and after baking. The doughs will be manufactured at the same consistency using different water absorption levels. Additionally, formulations will be gelatinized during kneading to verify the new protocol proposed.

## 2 Materials and methods

### 2.1 Materials

Chestnut (*Castanea sativa* Mill.) flour (surface mean diameter, D_s_, 35 ± 1 μm; water retention capacity, WRC, 3.8 ± 0.2 g water/g dry solid, d.b.) and seaweed powders of *Ascophyllum nodosum* (AN, D_s_, 77 ± 1 μm; WRC, 12.1 ± 0.1 d.b.), *Bifurcaria bifurcata* (BB, D_s_: 106 ± 5 μm; WRC: 14.4 ± 0.6 d.b.) and *Fucus vesiculosus* (FV, D_s_: 133 ± 5 μm; WRC: 10.5 ± 0.2 d.b.) brown seaweeds, guar gum (CAS: 9000-30-0, Sigma Aldrich), salt (CAS 7647-14-5, Sigma Aldrich) and tap water were employed as raw materials.

Chestnut flour chemical composition (% d.b.) was previously reported [15]: protein (6.3); fibre (3.8); starch (64.4); sugar (23.7) and fat (1.8). In the case of brown seaweeds the proximate composition is: carbohydrates: ≈52, 37 and 48; ash: ≈21.5, 34.0 and 17.5; lipids: ≈ 3.5, 6.0 and 5.0; proteins: ≈ 6, 11 and 10 and others ≈17, 12 and 19 for *Ascophyllum nodosum* [16], *Bifurcaria bifurcata* [17], and *Fucus vesiculosus* [18], respectively.

The determination of the particle size distribution of chestnut flours and seaweed powders was performed using different standard sieves (500, 250, 200, 125, 80, 63 and 40 μm, Cisa Industrial, Spain). Particles were passed through the different sieves and the corresponding fraction of particles retained in each sieve were weighed to obtain the corresponding mass fraction associated to each particle size. D_s_ was calculated by means of Eq. (1):

(1) $$D_{s}=\frac{1}{\sum_{i=1}^{n}\frac{x_{i}}{D_{pi}}}$$

where D_pi_ (μm) is the mean diameter for each fraction and x_i_ (-) is the mass fraction calculated as the ratio between corresponding mass of each fraction and the total mass of flour/powder employed in the sieving. The water retention capacity, WRC, of chestnut flour and seaweed powder was determined following an established protocol [19].

Different formulations were assayed. Control formulation consisted of chestnut flour, guar gum (2% flour basis, f.b.), salt (1.8% f.b.) and tap water. Seaweed enriched formulations consisted of blends based on the control formulation and seaweed powders AN, BB and FV added at different levels (3, 6 and 9% f.b.).

### 2.2 Dough processing

Gluten-free flour doughs were obtained after mixing the components in Mixolab^®^ apparatus employing two different methods. The first one consisted on mixing each formulation in order to obtain doughs with a target consistency of 1.10 ± 0.07 N m (C1) varying the amount of water added according to the standard protocol ICC-Standard Method No. 173 (2008). This protocol adjusts the initial moisture content of all formulations to 14% dry basis for comparative purposes. The total amount of flour (or flour + seaweed powder) and water placed into Mixolab^®^ bowl is 75 g and different hydrations of doughs are tested in order to obtain the target consistency. The water added defines the water absorption of each dough (WA, % f.b.) which is defined as quantity of water added to 100 g of flour (with 14% of moisture content) to form dough with the target consistency. Different doughs were obtained after the application of the protocol at constant consistency, C1, according to the employed formulation: control dough (CD) and *Ascophyllum nodosum* (CANX), *Bifurcaria bifurcata* (CBBX) and *Fucus vesiculosus* (CFVX) enriched doughs, where X corresponds to the level (3, 6 or 9% f.b.) of seaweed powder added. The second type consisted on the application of a hot and short mixing step (gelatinization protocol) to obtain seaweed powder enriched and non-enriched gelatinized-starch chestnut flour doughs (GCD, GCANX, GCBBX and GCFVX). This procedure consisted on three steps at 80 rpm: *i*) mixing during 5 min at 50 °C *ii*) heating (10 min) from 50 to 90 °C at 4 °C min^-1^ and iii) mixing during 4 min at 90 °C.

### 2.3 Thermo-mechanical analysis (DMTA)

Doughs at constant consistency were tested in a controlled stress rheometer (MCR 301, Anton Paar Physica, Austria) equipped with a chamber (CTD 450, Anton Paar Physica, Austria) using parallel plates (50 mm diameter, 2 mm gap). The thermomechanical assays were performed in the linear viscoelastic region of the doughs (0.1% of strain, 1 Hz of frequency). Temperature increased from 30 to 180 °C with a constant heating rate of 4 °C min^-1^. The rim of the samples was coated with paraffin (Panreac, Spain) to prevent water evaporation. All assays were performed at least in duplicate.

Storage modulus (G′) values were employed to determine the temperatures associated with thermal transitions using Rheoplus/32 software (version3.21, Anton Paar, Ostfildern, Germany). Moreover, SciDAVis 1.D013 free software was employed to carry out the mathematical treatment of DMTA data.

### 2.4 Doughs baking

The doughs prepared with the gelatinization protocol showed better moulding properties in relation to the doughs obtained with standard protocol. After kneading, samples were cooled at room condition up to ≈30 °C. Subsequently, they were manually moulded, cut in disks of 34 mm diameter and 3.6 mm height and scarified. Based on preliminary assays, doughs were baked in an oven (Balay 3HB569XC, BSH) at 180 °C during 25 min to produce cookies.

### 2.5 Antioxidant activity and polyphenols content

Samples of flour with seaweeds and milled cookies were submitted to a extraction operation and extracts were characterized by means of polyphenols (TP) and total solid (TS) content and DPPH scavenging activity (SA).

Extraction was assisted with an ultrasound processor (Hielscher, UIP-1000 hdT, Germany). All experiments were carried out in batch, the procedure starting with a 15 min rehydration step before extraction. Then extraction operation took place using a 200 mL beaker at controlled temperature (<35 °C) employing a cold water bath to avoid that temperatures increased could affect antioxidant activity. All extractions were performed using acetone-water (70:30 v/v) solution as solvent to obtain reference extracts [20]. The equipment operated with a frequency of 20 kHz and the irradiation power (<1000 W) was regulated in the ultrasound generator at 80% amplitude. The employed conditions were 4 min of contact time and 30 w/w of liquid-solid ratio. Finally, obtained extracts were centrifuged (≈12000 g for 15 min) using a high speed laboratory centrifuge (2–15, Sigma, UK) and the supernatant obtained was then filtered by pressure using syringe filters with a pore size of 0.25 μm and used for characterization analysis.

TP was measured as phloroglucinol (PHL) equivalents following a colorimetric method [21]. TP was evaluated in reference to raw seaweed powder sample (mg PHL/100 g dry sample, TP_w_) and also to total solids content in the extract (mg PHL/100 g dry solids, TP_s_). TS content in the extracts was determined after sample drying at 104 ± 1 °C. Samples were weighed daily until constant weight was reached after two consecutive measurements [22]. The SA assay measures the capacity of a system to react with a free radical agent (2,2-diphenyl-1-picrylhydrazyl, DPPH), employing a method previously proposed [23].

### 2.6 Statistical analysis

Experimental data were statistically analysed. Differences among means were identified by one-factor analysis of variance (ANOVA), followed by the Scheffé test and considering significant P-values ≤ 0.05 (IBM SPSS Statistics 22).

## 3 Results and discussion

### 3.1 Dough processing

Water absorption (WA) values of doughs to obtain the desired consistency (C1) are shown in Table 1. In general, seaweed powder addition to control dough (a flour blend consisting of chestnut flour + salt + guar gum, CD) required larger amounts of water to be added. WA of CD was 56.4 ± 0.8% d.b. which is in the range to those observed for wheat [24] or other gluten-free matrices like rice [25]. Specifically, WA varied as function of the type of seaweed powder. Concerning AN, WA significantly increased for doughs with >6% of seaweed powder addition (CAN6 and CAN9, >63.0%). BB addition had the strongest influence on WA of all seaweed powders. In fact, 3% of seaweed powder addition (CBB3) produced a significantly increase in WA (62.5 ± 0.9%) and CBB9 had the highest WA of all assayed doughs (70.8 ± 0.8%). Finally, FV addition influenced less the WA and this parameter was clearly higher to those of control formulation only at high amounts of seaweed powder (CFV9) added (64.0 ± 0.1%), representing the lowest WA value for a dough enriched with 9% of seaweed powder between the tested seaweeds. Although WA was significantly modified by seaweed powder addition, the WA values obtained were in the range of other gluten-free doughs such as wholegrain buckwheat or amaranth flour [26] and wheat flour doughs enriched with red seaweed (*Kappaphycus alvarezii*) powder incorporated (from 2 to 8%), whose WA varied even in a wider range from 58.5 ± 0.1 to 77.6 ± 0.2% [27]. The increase of WA of doughs with seaweed powder addition could be explained by higher water affinity of seaweed powders compared to chestnut flour. This can be observed in their different WRC. The larger WRC of seaweed powders (>10.5 g water/g dry solid) compared to chestnut flour (3.8 g water/g dry solid) clearly explain the found results.

**Table 1:** Onset (T_0_), peak (T_p_) and final (T_f_) temperatures of thermal transitions determined by DMTA following the elastic modulus (G′) of chestnut flour doughs (CD) and doughs from blends of chestnut flour and seaweed powders *Ascophyllum nodosum* (CANX), *Bifurcaria bifurcata* (CBBX) and *Fucus vesiculosus* (CFVX) (where X corresponds to the level (3, 6 or 9% f.b.) of seaweed powder added) obtained employing the standard protocol of mixing.∗

		CD	CAN3	CAN6	CAN9	CBB3	CBB6	CBB9	CFV3	CFV6	CFV9
WA (%, f.b.)		56.4 ± 0.8^a^	58.3 ± 0.6^a^	63.2 ± 0.3^b^	68.7 ± 0.5^c^	62.4 ± 0.7^b^	67.5 ± 1.3^c^	70.4 ± 1.0^d^	58.9 ± 0.7^a,b^	60.5 ± 1.0^b^	64.0 ± 0.5^c^
G	T_0_' (°C)	60.2 ± 1.0^a^	59.0 ± 0.9^a^	61.4 ± 0.1^a^	62.5 ± 1.5^a^	60.2 ± 1.1^a^	58.7 ± 0.5^a^	59.6 ± 0.1^a^	59.0 ± 1.4^a^	59.2 ± 0.7^a^	60.7 ± 1.6^a^
	T_0_ (°C)	71.5 ± 0.3^a^	70.7 ± 0.1^a^	70.3 ± 0.2^a^	69.9 ± 0.7^a^	71.3 ± 0.1^a^	68.8 ± 0.6^b^	68.2 ± 0.1^b^	70.0 ± 0.4^b^	69.4 ± 0.1^b^	69.6 ± 0.1^b^
	T_p_ (°C)	75.2 ± 0.3^a^	73.7 ± 0.2^b^	73.7 ± 0.4^b^	72.6 ± 0.1^b^	75.1 ± 0.1^a^	72.9 ± 0.3^b^	72.5 ± 0.1^b^	73.2 ± 0.4^b^	73.1 ± 0.1^b^	73.3 ± 0.1^b^
	T_f_ (°C)	79.1 ± 0.4^a^	77.6 ± 0.1^b^	77.4 ± 0.5^b^	76.9 ± 0.1^b^	78.9 ± 0.4^a^	76.6 ± 0.3^b^	76.0 ± 0.4^b^	77.1 ± 0.1^b^	76.8 ± 0.2^b^	77.1 ± 0.1^b^
M1	T_0_ (°C)	85.8 ± 0.4^a^	83.9 ± 0.6^b^	82.7 ± 0.2^b,c^	82.1 ± 0.1^c^	84.6 ± 0.1^b^	82.1 ± 0.1^c^	82.0 ± 0.1^c^	83.7 ± 0.4^b^	82.9 ± 0.4^b^	83.1 ± 0.1^b^
	T_p_ (°C)	88.3 ± 0.5^c^	87.8 ± 1.0^c^	92.5 ± 0.1^b^	96.6 ± 0.1^a^	91.1 ± 0.5^b^	91.4 ± 0.8^b^	91.6 ± 0.1^b^	92.9 ± 0.1^a^	92.6 ± 0.6^a^	91.4 ± 1.9^a^
	T_f_ (°C)	93.3 ± 1.4^b^	94.6 ± 0.2^a,b^	98.3 ± 2.1^a^	101.6 ± 0.5^a^	94.8 ± 0.4^b^	94.8 ± 1.9^b^	94.6 ± 0.5^b^	97.1 ± 0.1^b^	96.4 ± 0.6^b^	94.8 ± 1.6^b^
M2	T_0_ (°C)	115.8 ± 1.1^b^	122.0 ± 1.7^a^	123.8 ± 0.8^a^	125.9 ± 1.0^a^	120.8 ± 2.8^b^	120.9 ± 0.6^b^	120.1 ± 0.2^b^	125.1 ± 0.8^a^	121.2 ± 0.8^a^	123.0 ± 0.7^a^
	T_p_ (°C)	118.7 ± 1.6^b^	122.7 ± 1.8^a,b^	124.6 ± 1.2^a,b^	126.6 ± 1.1^a^	121.8 ± 2.6^b^	121.4 ± 0.7^b^	120.6 ± 0.1^b^	126.2 ± 0.8^a^	121.6 ± 0.8^a^	123.6 ± 0.8^a^
	T_f_ (°C)	119.5 ± 1.8^b^	123.8 ± 1.4^a,b^	126.2 ± 1.1^a^	127.7 ± 1.0^a^	122.6 ± 3.3^b^	122.7 ± 0.7^b^	122.4 ± 0.5^b^	127.8 ± 1.1^a^	123.3 ± 1.1^a^	124.9 ± 0.6^a^
M3	T_0_ (°C)	141.6 ± 6.3^a^	136.0 ± 2.4^a^	135.9 ± 3.2^a^	134.6 ± 0.4^a^	135.6 ± 2.9^a^	134.5 ± 1.8^a^	139.1 ± 2.8^a^	133.5 ± 2.3^a^	139.7 ± 1.8^a^	134.2 ± 0.7^a^
	T_p_ (°C)	152.9 ± 5.0^a^	143.4 ± 0.4^a^	140.7 ± 5.4^a^	138.9 ± 1.1^a^	140.1 ± 3.2^a^	138.5 ± 3.0^a^	143.4 ± 2.3^a^	143.3 ± 3.0^a^	145.1 ± 3.9^c^	143.7 ± 4.1^a^
	T_f_ (°C)	156.0 ± 6.2^a^	149.7 ± 0.4^a^	147.4 ± 1.3^a^	152.5 ± 0.6^a^	145.1 ± 1.3^a^	144.0 ± 2.7^a^	147.3 ± 1.0^a^	150.7 ± 0.8^a^	151.3 ± 0.8^a^	149.2 ± 4.0^a^

∗ Data are presented as means ± standard deviation. Data value of each parameter with different superscript letters in rows (for each seaweed compared to control) are significantly different (P ≤ 0.05).

The different trends of WA as function of the employed seaweed powder could also be justified jointly by the WRC and the mean particle size of each seaweed powder. BB addition produced a higher influence on WA because of its higher WRC (14.4 g water/g dry solid) compared to those of AN and FV (<12.1 g water/g dry solid). The higher WA with AN addition in relation to FV could be related to the different particle size of these powders. AN powders had a significantly lower particle size compared to FV (D_s_: 77 *vs* 133 μm, respectively). Small particle sizes of powders are often related to high surface area of these systems that could enhance the water absorption rate.

### 3.2 Characteristic temperatures determination of transitions by DMTA

Chestnut flour (CD) and chestnut flour-seaweed powder (CANX, CBBX and CFVX) doughs were evaluated by DMTA to determine the starch transition peaks. In order to carry out this analysis, several minimum, maximum and inflection points of G′ *vs* temperature during temperature sweeps were determined. Table 2 summarizes the proposed protocol.

**Table 2:** Proposed determination protocol of onset (T_0_), peak (T_p_) and final (T_f_) characteristic temperatures of thermal transitions employing DMTA data following the elastic modulus (G′) for starchy doughs.

Transition		Determination	Figure
G	T_0_′	Minimum of G′(T)	1a
	T_0_	Inflexion point of G′(T), d^2^G'/dT^2^ = 0	2
	T_p_	Local maximum of G′(T)	2
	T_f_	Inflexion point of G′(T), d^2^G'/dT^2^ = 0	2
M1	T_0_	Inflexion point of G′(T), d^2^G'/dT^2^ = 0	2
	T_p_	Local minimum of d^2^G'/dT^2^*vs* T	2
	T_f_	Inflexion point of G′(T), d^2^G'/dT^2^ = 0	2
M2	T_0_	Inflexion point of G′(T), d^2^G'/dT^2^ = 0	3b
	T_p_	Local minimum of d^2^G'/dT^2^*vs* T	3b
	T_f_	Inflexion point of G′(T), d^2^G'/dT^2^ = 0	3b
M3	T_0_	Inflexion point of G′(T), d^2^G'/dT^2^ = 0	4
	T_p_	Local minimum of d^2^G'/dT^2^*vs* T	4
	T_f_	Inflexion point of G′(T), d^2^G'/dT^2^ = 0	4

#### 3.2.1 G transition

**Fig. 1:**
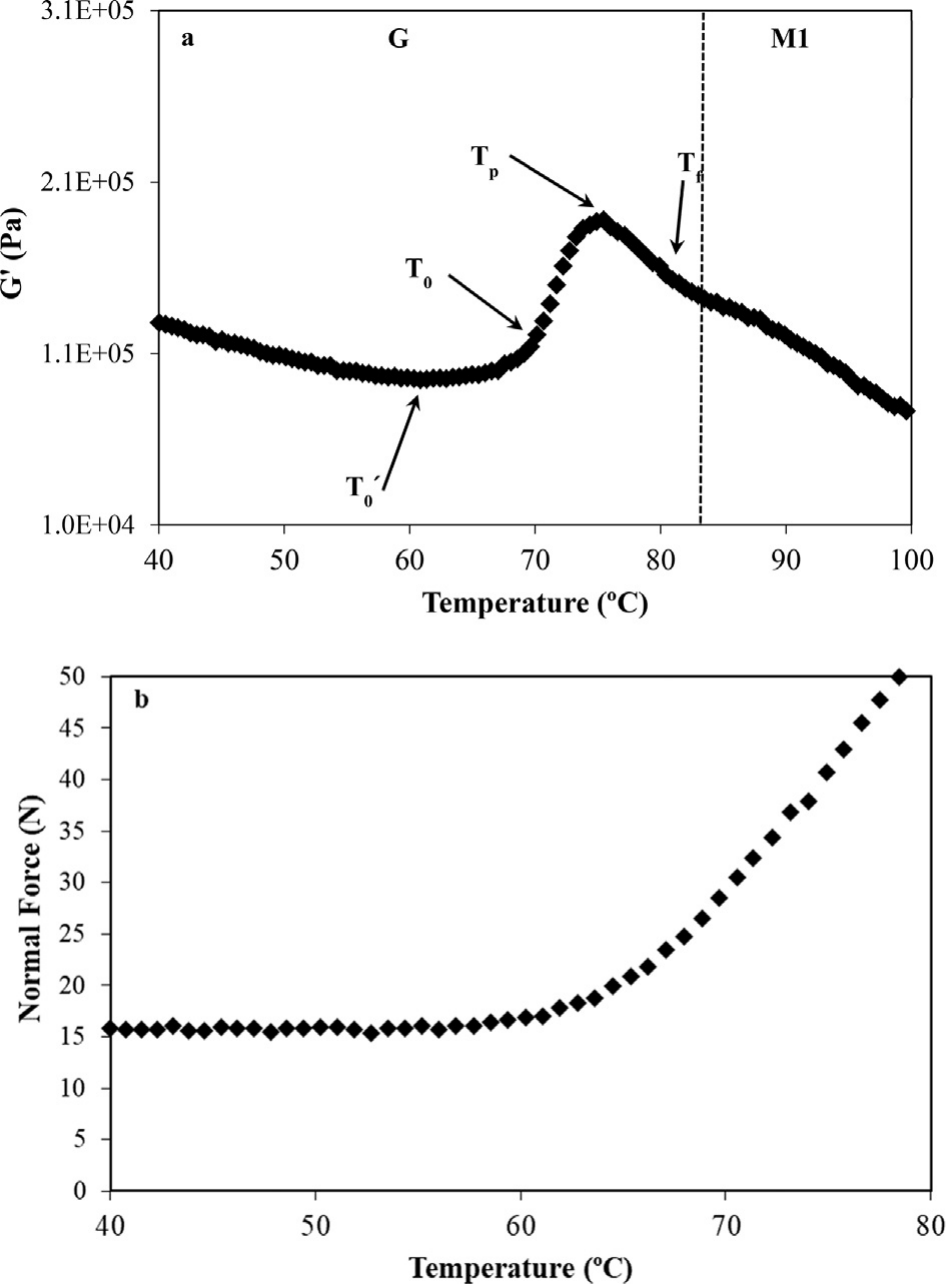
Experimental data of G′ *vs* temperature (a) and normal force *vs* temperature (b) for CD during gelatinization (G) employing DMTA analysis.

Fig. 1a shows the G′ peaks during heating of CD, as example of starch gelatinization transition of doughs. Applying the same method described by [28] different characteristics temperatures of G + M1 transition can be determined. At low temperatures G′ values decreases slightly with increasing temperature due to proteins weakening up to achieve a minimum, Fig. 1a. This point is labelled like T_0_', Table 1, and determines the beginning of the physical phenomena that take place during starch gelatinization, mainly the swelling of the starch granules. This process can be clearly observed in Fig. 1b where the data of dough normal force applied to the plates of rheometer *vs* temperature during DMTA analysis of CD are plotted. As it can be observed, at temperatures closer to T_0_′ the normal force of the dough between the plates increased, indicating a trend of dough to increase its volume due to swelling of starch granules. This point is not detected during DSC experiments, because the involved processes do not modify significantly the thermal properties of the system [28].

**Fig. 2:**
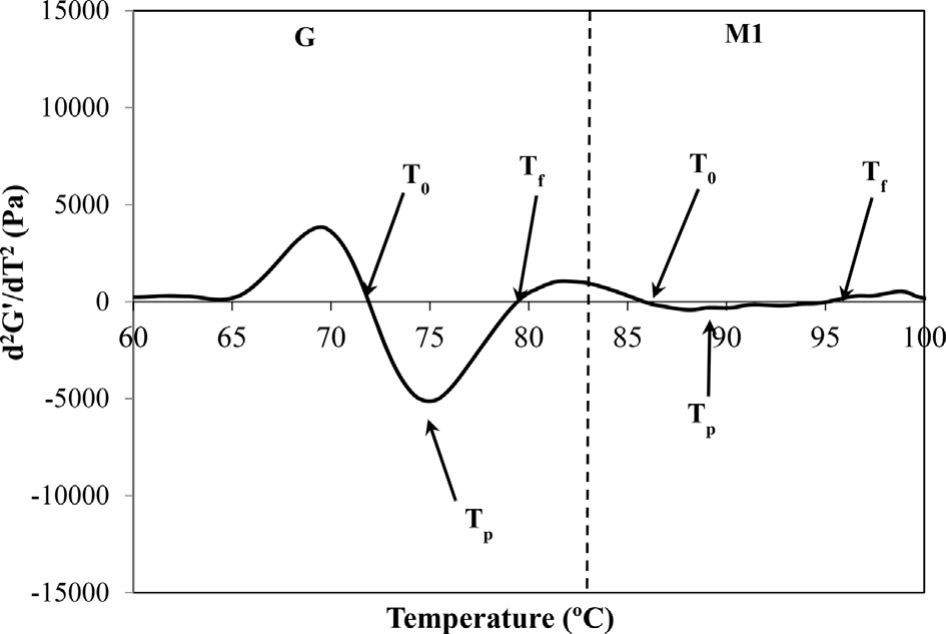
Second derivative of G′(T) (d^2^G'/dT^2^) for DMTA experimental data of CD, G and M1 transition.

After T_0_′, G′ increases due to the growing turgor of starch granules. Nevertheless, starch gelatinization is also originated by the disintegration of the granules and starchy polymers melting with a generation of a continuous matrix of leached amylose molecules that increases the viscosity and consequently the viscous character of the multi-phased system [29]. The beginning of this process was usually measured by DSC due to it involved thermal changes in the sample and labelled as T_0_ [6]. However, it could be also determined employing DMTA technique. It corresponded to inflexion point of G′ as it can be observed in Fig. 1a. Moreover, as T_0_ corresponds to an inflexion point, it can be also determined by using the mathematical definition of inflection point (d^2^G'/dT^2^ = 0), Fig. 2. This plotting enables the determination of T_0_ easily.

The peak temperature of G transition, T_p_, can be determined by means of two procedures: a) the relative maximum of G' (Fig. 1a) or b) the local minimum of d^2^G'/dT^2^ *vs* T (Fig. 2).

Final temperature, T_f_, can be evaluated (sometimes with difficulty) through the inflexion point of G′(T) curve (the point in which the slope of G' (Fig. 1a) changes after T_p_ [28]. As in the case of T_0_, a clearer way is to determine T_p_ at the point where d^2^G'/dT^2^ = 0 (Fig. 2).

#### 3.2.2 M1 transition

Sometimes M1 transition is overlapped by G transition which makes difficult the determination of their characteristics temperatures. Depending on the nature of the starch source and hydration level of dough, the determination of both transitions G and M1 could be carried out employing DSC technique [28]. However, up to our knowledge, no methods to determine T_0_ and T_p_ of M1 transition employing DMTA technique even if it is non-overlapped by G transition can be found in the literature. In this work it is proposed a new method of determination of these characteristics temperatures of M1 transition by DMTA technique. In this sense, T_0_ can be determined as the inflexion point of G′(T) (d^2^G'/dT^2^ = 0) at temperatures higher than T_f_ of G transition, T_p_ as the local minimum of the same curve and T_f_ also as the inflexion point of G′(T) (d^2^G'/dT^2^ = 0), at higher temperatures than T_p_ of M1 transition, Fig. 2.

#### 3.2.3 M2 transition

**Fig. 3:**
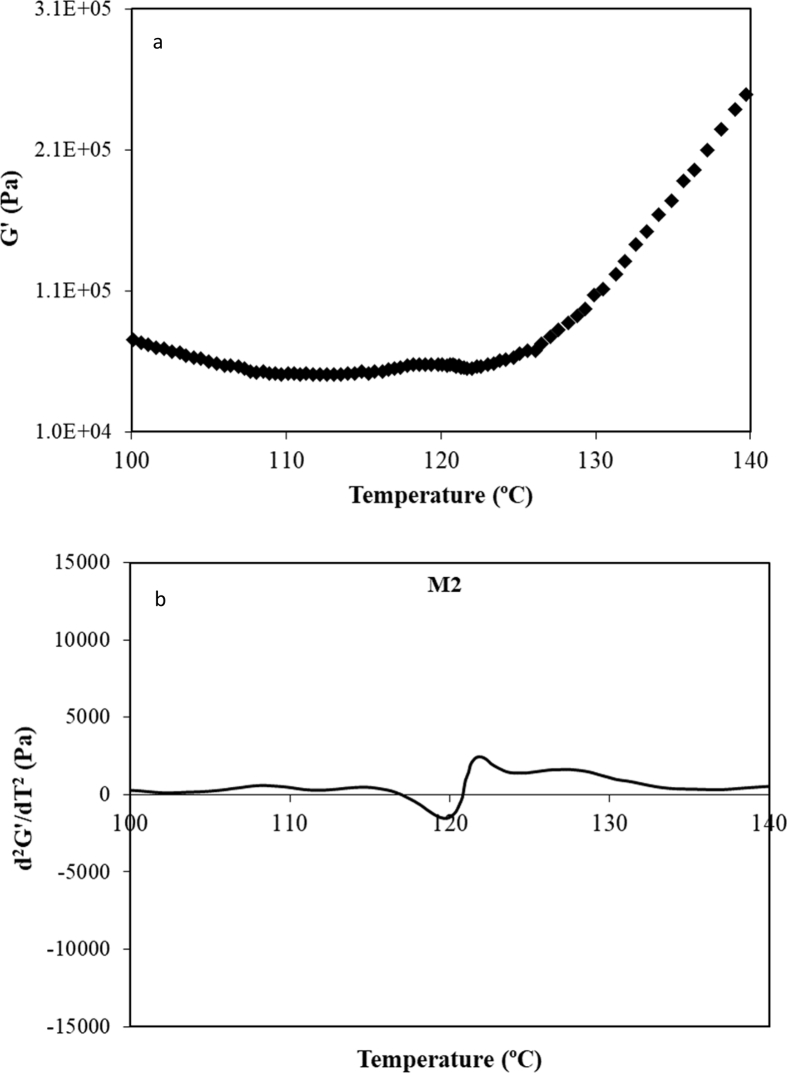
Experimental data of G′ *vs* temperature (a) and second derivative of G′(T) (d^2^G'/dT^2^) for DMTA experimental data of CD during M2 transition.

M2 peak is difficult to observe because is coincident with the water evaporation from the dough sample. After M1 transition G′ values decrease until a minimum and then continue to increase, Fig. 3a. In these circumstances, simultaneous processes that affect to mechanical properties take place and the G′ data show some dispersion due to the important physical and structural changes (matrix porosity) promoted by the water removal [30]. Regarding this transition, T_p_ is calculated as the local minimum of d^2^G'/dT^2^ *vs* T. T_0_ and T_f_ can be determined by means of inflexion point of G′(T) (d^2^G'/dT^2^ = 0) before and after T_p_, respectively (Fig. 3b).

#### 3.2.4 M3 transition

**Fig. 4:**
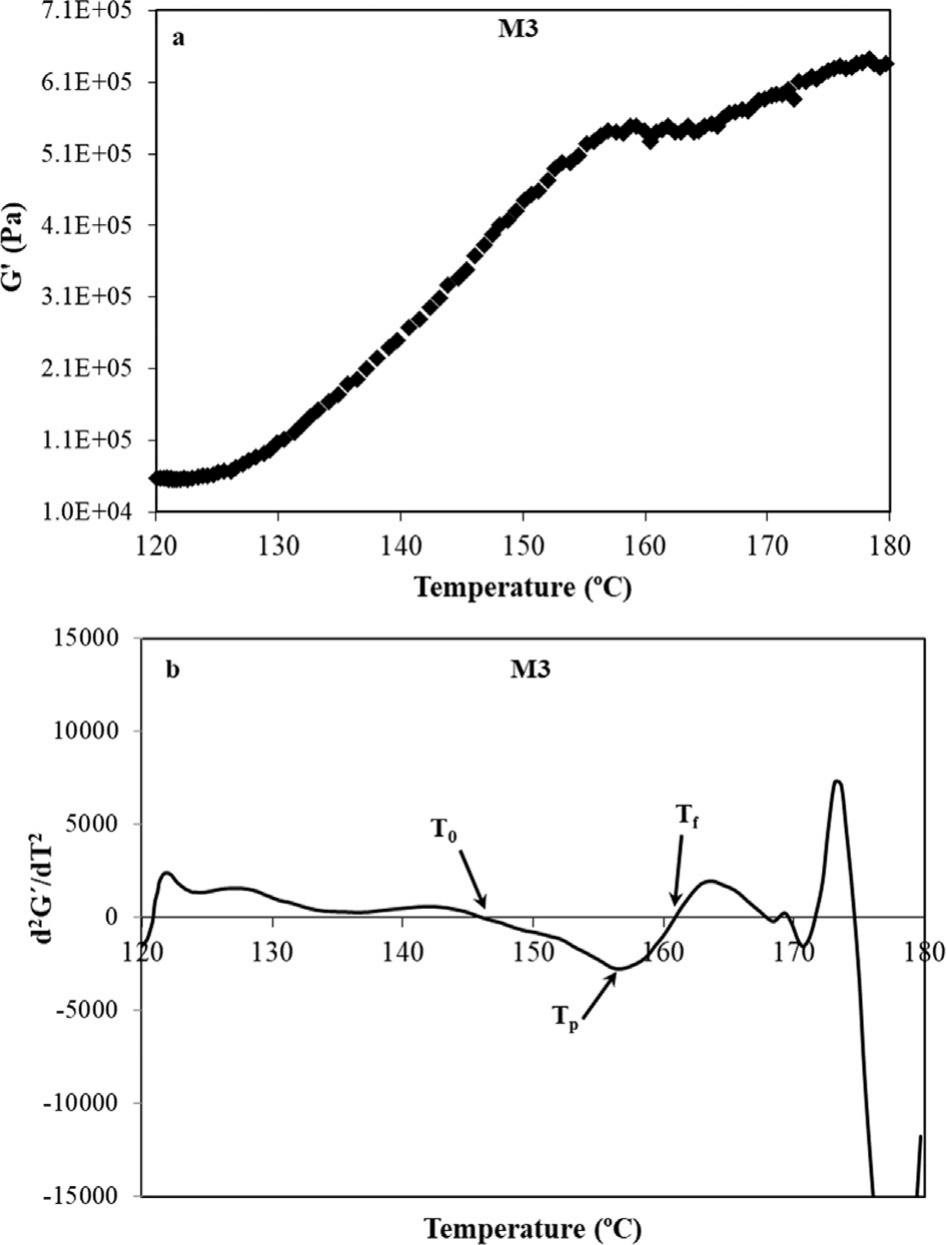
Experimental data of G′ *vs* temperature (a) and second derivate of G′(T) (d^2^G'/dT^2^) for DMTA experimental data of CD during M3 transition.

Above M2 transition, G′ values increase sharply during baking by the complex phenomena related to the crust formation that give as result a more rigid and stiff material, Fig. 4a. Regarding to M3 peak, corresponding to the melting of amylose, it can be monitored by G′ trend considering the curve obtained from its second derivative (d^2^G'/dT^2^) *vs* T. T_0_ and T_f_ can be determined by the corresponding inflexion points of the G′(T) (d^2^G'/dT^2^ = 0) whilst T_p_ can be also evaluated as the local minimum of d^2^G'/dT^2^ = 0 *vs* T, Fig. 4b. M3 peaks determined by DMTA are, in general, broader than those obtained by DSC due to the associated structural changes and rearrangements of the transitions take place in an extensive range of temperatures [28].

At temperatures higher than 170 °C, no transitions could be found mainly due the lack of reproducibility of experimental data.

### 3.3 Application of the proposed method of thermal transitions determination on chestnut flour and seaweed-enriched chestnut flour doughs

The thermal transitions of chestnut flour and seaweed-enriched chestnut flour doughs were evaluated employing the protocol previously developed, Table 2, to carry out the analysis of characteristic temperatures of thermal transitions on gluten-free flour doughs by DMTA, Tables 1 and 3.

**Table 3:** Onset (T_0_), peak (T_p_) and final (T_f_) temperatures of thermal transitions determined by DMTA following the elastic modulus (G′) of doughs gelatinized during mixing obtained from chestnut flour (GCD) and blends of chestnut flour and seaweed powders *Ascophyllum nodosum* (GCANX), *Bifurcaria bifurcata* (GCBBX) and *Fucus vesiculosus* (GCFVX) (where X corresponds to the level (3, 6 or 9% f.b.) of seaweed powder added).∗

		GCD	GCAN3	GCAN6	GCAN9	GCBB3	GCBB6	GCBB9	GCFV3	GCFV6	GCFV9
M1	T_0_ (°C)	88.0 ± 0.3^a^	92.0 ± 2.0^a^	89.2 ± 1.9^a^	86.0 ± 0.3^b^	88.2 ± 1.0^a^	79.9 ± 0.3^b^	83.6 ± 0.1^b^	85.7 ± 0.5^a^	86.9 ± 1.9^a^	89.9 ± 2.0^a^
	T_p_ (°C)	92.4 ± 0.6^a^	95.5 ± 1.4^a^	92.8 ± 1.4^a^	91.5 ± 0.4^a^	92.8 ± 0.4^a^	90.5 ± 1.6^a^	87.7 ± 0.7^a^	89.4 ± 0.2^a^	92.6 ± 1.7^a^	93.5 ± 1.2^a^
	T_f_ (°C)	95.2 ± 1.0^a^	97.1 ± 0.6^a^	95.9 ± 1.3^a^	94.6 ± 2.0^a^	95.3 ± 0.3^a^	94.0 ± 1.6^a^	92.4 ± 0.0^a^	94.3 ± 2.8^a^	95.5 ± 1.3^a^	96.1 ± 0.5^a^
M2	T_0_ (°C)	108.2 ± 0.3^a^	108.1 ± 0.1^a^	108.5 ± 0.4^a^	108.5 ± 0.4^a^	107.9 ± 0.8^a^	108.9 ± 0.3^a^	107.2 ± 0.1^a^	107.4 ± 1.2^a^	106.9 ± 0.6^a^	108.6 ± 0.1^a^
	T_p_ (°C)	111.3 ± 0.4^a^	111.4 ± 0.2^a^	111.1 ± 0.2^a^	111.0 ± 0.3^a^	110.9 ± 0.6^a^	111.0 ± 0.1^a^	110.6 ± 0.4^a^	110.3 ± 0.8^a^	108.9 ± 3.5^a^	111.5 ± 0.1^a^
	T_f_ (°C)	112.1 ± 0.2^a^	112.3 ± 0.2^a^	111.9 ± 0.2^a^	111.8 ± 0.4^a^	112.1 ± 0.8^a^	112.2 ± 0.3^a^	111.4 ± 0.5^a^	111.1 ± 0.6^a^	111.4 ± 1.3^a^	112.5 ± 0.4^a^
M3	T_0_ (°C)	156.9 ± 3.2^a^	157.4 ± 1.1^a^	158.5 ± 1.5^a^	158.2 ± 0.6^a^	153.2 ± 0.2^a^	153.7 ± 0.5^a^	153.7 ± 2.1^a^	156.5 ± 0.1^a^	155.1 ± 3.4^a^	154.6 ± 1.3^a^
	T_p_ (°C)	164.7 ± 2.9^a^	163.9 ± 2.6^a^	163.7 ± 0.3^a^	166.1 ± 1.8^a^	158.7 ± 1.3^a^	159.0 ± 0.1^a^	157.8 ± 2.5^a^	161.7 ± 0.4^a^	159.3 ± 5.8^a^	160.5 ± 0.8^a^
	T_f_ (°C)	170.6 ± 0.1^a^	170.9 ± 0.1^a^	171.5 ± 0.0^a^	171.0 ± 0.1^a^	167.9 ± 4.9^a^	166.4 ± 6.2^a^	169.7 ± 1.9^a^	169.3 ± 3.2^a^	163.7 ± 5.9^a^	165.5 ± 0.6^a^

∗ Data are presented as means ± standard deviation. Data value of each parameter with different superscript letters in rows (for each seaweed compared to control) are significantly different (P ≤ 0.05).

#### 3.3.1 Seaweed-enriched chestnut flour doughs

Regarding gelatinization process (G + M1 transition), both G and M1 transitions could be determined separately. Concerning G transition, it was observed that no significant differences existed on the corresponding temperature of the beginning of swelling of starch granules (T_0_′). This fact seems to indicate that, although larger WA values were necessary to obtain doughs with similar consistency when seaweed powders were added, Table 1, the water availability for the chestnut starch granules present in flours was the same in all assayed doughs. In other words, the additional amount of water to obtain the dough when seaweed powders are present seems to interact directly with seaweed powder particles and not with the starch favouring the swelling of these particles at lower temperatures.

**Fig. 5:**
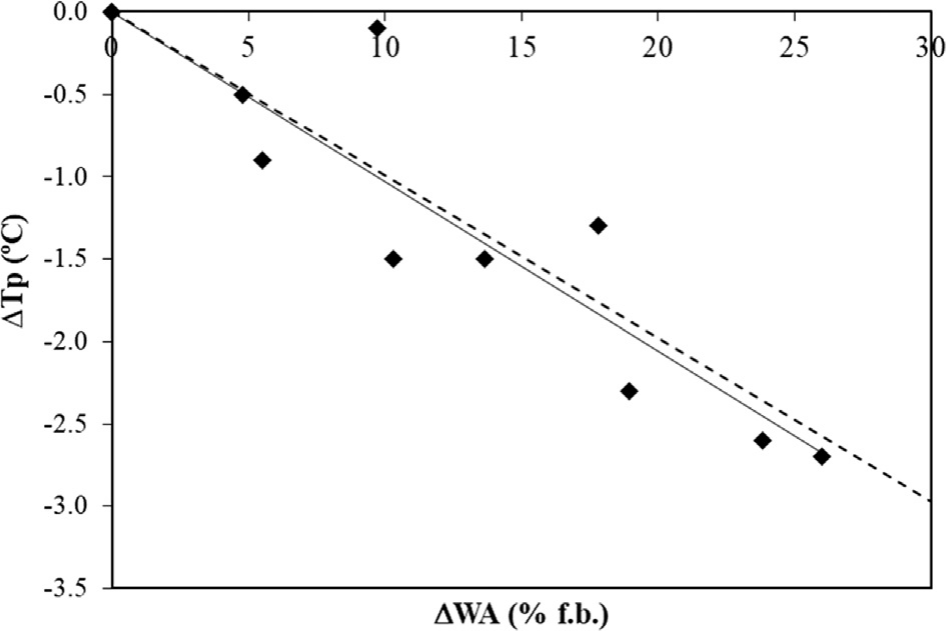
Relationship between WA increase and T_p_ decrease for seaweed-enriched chestnut-flour doughs (♦) and chestnut starch dispersions (- - -, [31]).

On the other hand, the corresponding temperature of disintegration of the starch granules and starchy polymers melting (T_0_) seems to be significantly decreased by seaweed powder addition in the case of doughs enriched with BB (≥6%) and FV (≥3%), Table 1. A similar behaviour was observed for T_p_ and T_f_ of all studied seaweed-enriched doughs. This behaviour could be due to larger WA absorption of these doughs. Due to swelling there is an increase in the turgor of starch granules that could lead to a higher pressure against the hydrated seaweed particles. This fact could promote the water leaching from seaweed particles to dough matrix and consequently increases the available water facilitating the starch granules melting and hence leads to a decrease on the characteristic temperatures values. In fact, it was observed that the effect of WA on T_p_ values of seaweed-enriched chestnut flour doughs is the same of those observed for chestnut starch dispersions at different hydration levels [31]. In Fig. 5, it can be observed the relationship between WA increase and T_p_ decrease for seaweed-enriched chestnut flour doughs and chestnut starch dispersions. This result allows the conclusion that seaweed powder addition only modified the physical process of starch swelling in doughs, avoiding the well-known decrease of T_0_' with increasing WA due to the high WRC of seaweed particles.

In the case of M1 transition, it was observed that seaweed powder addition significantly reduced T_0_ values for all studied doughs, following the same trend previously described for G transition. In all cases, T_0_ decreased from ≈86 °C for control dough to ≈82 °C for samples with seaweed powders. However, T_p_ and T_f_, increased with seaweed powder addition. G′ decreases at this temperature range and again a competition for available water, after partial starch granule structure disintegration, between seaweed particles and starch takes place. Higher WRC of seaweed in comparison to chestnut flour could reduce the available water for the starch on seaweed-enriched flours and hence both characteristic temperatures increase. This hypothesis is corroborated by the fact that samples with AN (powders with lower average particle sizes and consequently higher number of particles added and total surface area developed) addition showed higher variations of both temperature values. In fact, T_f_ did not significantly varied for doughs enriched with BB and FV and increased up to 101.6 °C in the case of doughs enriched with AN.

M2 transition was observed in all flour doughs. It varied between ≈115 to ≈128 °C and the characteristic temperature associated of the beginning of this process was significantly increased by seaweed powder addition in doughs and with similar trends (and explanations) of the delay of the transition that in the case of M1 transition. Moreover [17], reported that brown seaweeds have a relevant percentage of lipids which could led to expect that some interactions between seaweed-lipids and amylose of flour could occur and hence the beginning of this process be retarded. However, the fact that this transition ends at the same temperature for all assayed seaweeds, seems to indicate that these interactions, in the case that they occur, they did not form amylose-lipids complex more resistant to temperature, probably due to the high insolubility of seaweed particles (>80%) that makes difficult these deeper interactions. The temperature range observed was similar of those previously reported in literature [32, 33].

Finally, the temperature range of M3 transition varied from 134 to 156 °C, a typical range of temperatures to this transition, as previously reported by [34]. No significant differences were observed due to seaweed powder addition. Note here that at these temperatures, the G′ data show some dispersion between the duplicates which makes the results to have a larger standard deviation. Moreover, this transition only depends on amylose and at these temperatures the studied doughs presented low water content and hence no modifications on dough behaviour due to seaweed powder addition could be expected.

#### 3.3.2 Seaweed-enriched pregelatinized chestnut flour doughs

**Fig. 6:**
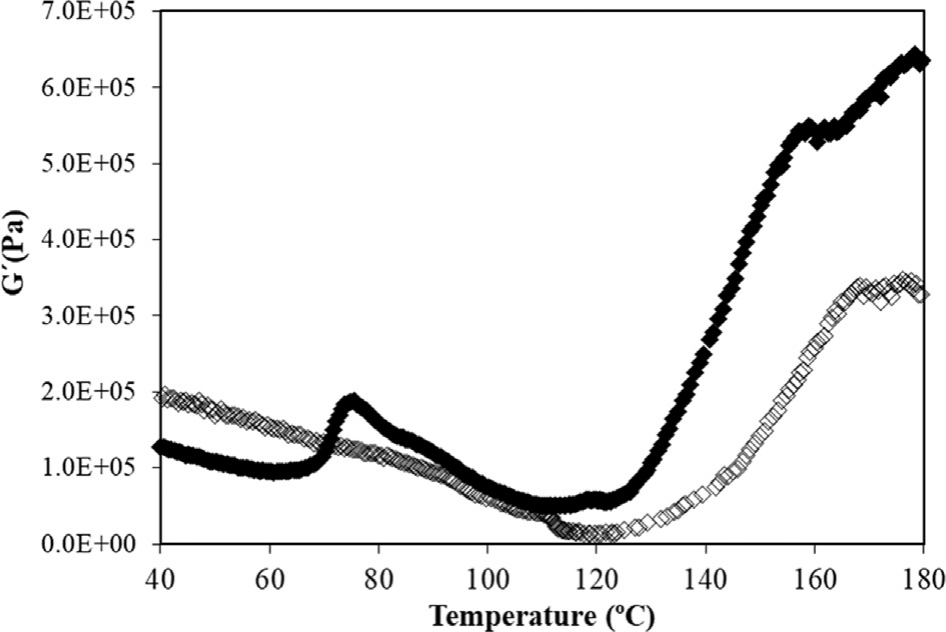
G′ evolution with temperature for gluten free chestnut flour doughs obtained employing standard protocol (♦) and gelatinization protocol of mixing (◊).

A comparison between thermal profiles of G′ for CD dough obtained with the standard mixing protocol and the one obtained with the gelatinization protocol using the same formulation (GCD) are presented in Fig. 6. As it can be seen, no transition associated to gelatinization (G) was observed. This fact confirmed that starch was gelatinized during mixing applying the new protocol. Applying the proposed protocol based on G′ trend with temperature previously explained the remaining transitions could be determined, Table 3. M1 was observed in all studied doughs indicating that although starch gelatinization was mainly performed during mixing step, some of amylopectin remained without melting. M2 (108–112 °C) and M3 (155–170 °C) were also observed in all studied doughs. Concerning M1 transition, a significant decrease on T_0_ was observed in some doughs enriched with AN and BB powders. This behaviour could be related to the higher initial WA of these doughs that resulted in higher hydration at the end of mixing step. These higher hydration levels of doughs enriched with AN and BB decrease the temperature of the beginning of amylopectin crystallites melting. In comparison with doughs obtained with the standard mixing protocol, it was observed a delay in the characteristic temperatures associated to this transition. This result could be explained by lower hydration of doughs at the beginning of DMTA test, due to some amount of water was evaporated during the previous mixing-gelatinization process. In the case of M3, it has to be noted that this transition was moved to higher temperatures compared to doughs obtained with the standard mixing protocol probably due to the same effect. It has been reported that initial WA could have an noticeable influence in this transition at higher temperatures [6] indicating that low initial WA increases the characteristic temperatures for this transition as it happens in this case.

### 3.4 Effect of baking on antioxidant properties of samples

Acetone/water extracts from blends of chestnut flour enriched with AN, BB and FV before and after baking (cookies) were analysed in terms of total polyphenolic content (TP_w_, TP_s_) and scavenging activity (SA), Table 4.

**Table 4:** Total polyphenolic (TP) and scavenging activity (SA) of acetone:water (70:30) extracts of chestnut flour (CD) and blends of chestnut flour with *Ascophyllum nodosum* (CANX), *Bifurcaria bifurcata* (CBBX) and *Fucus vesiculosus* (CFVX) seaweed powders (where X corresponds to the level (3, 6 or 9% f.b.) of seaweed powder added) before and after baking process.∗

	Before baking			After baking		
	TP_W_	TP_S_	SA	TP_W_	TP_S_	SA
CD	155 ± 16^d^	755 ± 77^d^	16.9 ± 3.1^c^	876 ± 71^a^	3025 ± 239^a^	75.9 ± 5.1^a^
CAN3	282 ± 14^c^	1389 ± 69^c^	24.7 ± 2.8^c^	926 ± 94^a^	3024 ± 308^a^	76.0 ± 0.7^a^
CAN6	472 ± 39^b^	2235 ± 182^b^	39.9 ± 4.7^a^	601 ± 113^b^	1699 ± 318^b^	52.8 ± 3.4^b^
CAN9	622 ± 34^a^	2804 ± 154^a^	48.6 ± 2.9^a^	430 ± 34^c^	1154 ± 91^c^	43.8 ± 1.7^b^
CBB3	206 ± 20^c^	927 ± 89^c^	18.5 ± 5.0^c^	769 ± 68^a,b^	1124 ± 99^c^	72.8 ± 1.8^a^
CBB6	320±7^b^	1461 ± 34^b^	30.8 ± 2.5^b^	716 ± 78^b^	2536 ± 276^b^	68.5 ± 2.1^a^
CBB9	419 ± 15^a^	1803 ± 65^a^	43.8 ± 1.1^a^	646 ± 57^b^	3175 ± 278^a^	70.6 ± 2.8^a^
CFV3	188±6^d^	867 ± 26^d^	16.0 ± 2.2^c^	740 ± 85^a,b^	1752 ± 201^b,c^	71.0 ± 1.4^a^
CFV6	249 ± 34^b^	1164 ± 157^b^	33.0 ± 7.4^a^	672 ± 79^b^	1375 ± 162^c^	70.3 ± 5.4^a^
CFV9	386 ± 14^a^	1664 ± 60^a^	37.9 ± 7.5^a^	746 ± 63^a,b^	1800 ± 151^b^	68.0 ± 2.8^a^

TP_w_ is referred to raw powder (mg PHL/100 g dry powder), TP_s_ is referred to total solids content in the extract (mg PHL/100 g dry solids) and SA as % of total inhibition after 1 h.

∗ Data are presented as means ±standard deviation. Data value of each parameter with different superscript letters in columns (for each seaweed compared to control) are significantly different (P ≤ 0.05).

The seaweed powder addition significantly increased TP of flour blends. Specifically, for AN and BB an addition of 3% significantly increased TP whilst for FV a minimum of 6% was necessary. In the case of SA, a minimum of 6% was necessary for all blends. However, for AN and FV, SA was not increased significantly when adding larger contents than 6%. In this context, based on SA results, the optimum value of seaweed addition before baking is 6% for chestnut flour blends enriched with AN or FV and 9% for BB. The largest SA values were obtained for flour blends enriched with AN followed by BB and FV. These results are in accordance with previous studies where AN aqueous extracts [35] showed higher SA and TP than FV [36].

A linear relationship was obtained between SA and both TP_w_ and TP_s_ (R^2^ > 0.9). This fact seems to indicate that antioxidant activity of flour blends is mainly related to their TP. A similar relationship between TP and SA was already reported for green and red seaweeds [37]. This result seems to indicate that once seaweed powders are added to chestnut flours the SA of chestnut flour blends are mainly defined by the antioxidant capacity of seaweed powder added and not to the chestnut flour antioxidant properties.

TP and SA values increased after baking, except in the case of addition of 9% AN. The increase of SA induced by baking is related to the Maillard's reactions. Previously, other authors reported that these Maillard's products are responsible of increase on TP [38] and antioxidant activity [39, 40] of bakery products. However, seaweed addition decreased TP values of samples in comparison with the control. This trend is the opposite of that previously described for the unbaked samples. This difference could be also explained by the formation of Maillard's reaction products [41]. reported that browning intensity is related to low moisture content. In the case of control sample the higher TP values could be due to the fact that their WA values are lower compared to the enriched doughs. Although during baking water is evaporated in all doughs, hygroscopic seaweed particles could aid to retain water during baking which could delay the Maillard reaction in seaweed-enriched baked doughs compared to control.

For SA, two different trends were observed. For chestnut doughs enriched with BB and FV, non-significant effect of addition was observed on SA values. Moreover, it has to be noted that, in both cases, the previously mentioned relationship of SA *vs* TP was lost. This fact leads to conclude that SA values achieved by these cookies were mainly due by reactive compounds generated during baking not measured as TP contrary to which it was observed for flour-seaweed powder blends before baking. However, in the case of samples enriched with AN, seaweed addition significantly decreased SA and the aforementioned relationship of SA with TP remained after baking, indicating that powder additions >3% of AN significantly decreased the antioxidant characteristics of cookies.

SA values achieved by cookies are not mainly related to their TP, as in the case of the flour-seaweed powder mixtures, but other reactive compounds generated during baking are responsible.

## 4 Conclusions

A protocol for the determination of characteristic temperatures of thermal transitions was satisfactorily tested employing DMTA on gluten-free flour doughs based on chestnut flours supplemented with brown seaweed powders (*Ascophyllum nodosum, Bifurcaria bifurcata and Fucus vesiculosus*). Results show that characteristic temperatures of starch gelatinization (G), melting of amylopectin crystallites (M1), amylose-lipid transition (M2) and amylose melting (M3) that take place in gluten-free flour doughs can be determined employing the proposed protocol of determination. These temperatures depend strongly on water absorption of starchy matrix. Characteristic temperatures were related to hygroscopic properties of seaweed particles (by means of water retention capacity) and particle size (by means of mean surface diameter) by its importance on the competition for available water. The study of gelatinized doughs confirmed that the proposed protocol adequately determine starch gelatinization. Authors consider that this protocol could be easily applied to other starchy systems of similar nature (with and without gluten). Finally, seaweed powder addition had a positive effect on antioxidant properties of doughs before baking. However, the influence of seaweed powder addition of baked products (cookies) is overlapped by Maillard's products generated during baking which makes the analysis more complex.

## Declarations

### Author contribution statement

Santiago Arufe: Designed the experiments; performed the experiments; Analysed and interpreted the data; Wrote the paper.

Jorge Sineiro, Ramón Moreira: Conceived and designed the experiments; Analysed and interpreted the data.

### Funding statement

This work was supported by the Ministry of Economy and competitiveness of Spain and European Regional Development Fund (ERDF) of European Union by the research project (CTQ 2013–43616/P).

### Competing interest statement

The authors declare no conflict of interest.

### Additional information

No additional information is available for this paper.
